# A randomized controlled trial of Goal Management Training for executive functioning in schizophrenia spectrum disorders or psychosis risk syndromes

**DOI:** 10.1186/s12888-022-04197-3

**Published:** 2022-08-28

**Authors:** Ingvild Haugen, Jan Stubberud, Elisabeth Haug, Susan R. McGurk, Kjell Tore Hovik, Torill Ueland, Merete Glenne Øie

**Affiliations:** 1 Division of Mental Health Care, Innlandet Hospital Trust, P. O. Box 104, 2381 Brumunddal, Norway; 2 Department of Psychology, University of Oslo, P.O. Box 1094, 0317 Oslo, Norway; 3 Department of Research, Lovisenberg Diaconal Hospital, P.O. Box 4970, Nydalen, 0440 Oslo, Norway; 4 Departments of Occupational Therapy and Psychological and Brain Sciences, Boston University, 930 Commonwealth Avenue, Boston, MA 02215 USA; 5 Department of Psychology, Inland Norway University of Applied Sciences, P.O.Box 400, Elverum, Norway; 6 Norwegian Centre for Mental Disorders Research, Oslo University Hospital, Postboks 4956, Nydalen, 0424 Oslo, Norway; 7 Research Division, Innlandet Hospital Trust, P.O. Box 104, 2381 Brumunddal, Norway

**Keywords:** Early intervention, Psychosis, Executive function, Cognitive remediation, Cognitive impairment, Real-world function

## Abstract

**Background:**

Executive functioning is essential to daily life and severely impaired in schizophrenia and psychosis risk syndromes. Goal Management Training (GMT) is a theoretically founded, empirically supported, metacognitive strategy training program designed to improve executive functioning.

**Methods:**

A randomized controlled parallel group trial compared GMT with treatment as usual among 81 participants (GMT, *n* = 39 versus Wait List Controls, *n* = 42) recruited from an early intervention for psychosis setting. Computer generated random allocation was performed by someone independent from the study team and raters post-intervention were unaware of allocation. The primary objective was to assess the impact of GMT administered in small groups for 5 weeks on executive functioning. The secondary objective was to explore the potential of the intervention in influencing daily life functioning and clinical symptoms.

**Results:**

GMT improved self-reported executive functioning, measured with the Behavior Rating Inventory of Executive Function – Adult version (BRIEF-A), significantly more than treatment as usual. A linear mixed model for repeated measures, including all partial data according to the principle of intention to treat, showed a significant group x time interaction effect assessed immediately after intervention (post-test) and 6 months after intervention (follow-up), *F* = 8.40, *p* .005, *r* .37. Improvement occurred in both groups in objective executive functioning as measured by neuropsychological tests, functional capacity, daily life functioning and symptoms of psychosis rated by clinicians. Self-reported clinical symptoms measured with the Symptoms Check List (SCL-10) improved significantly more after GMT than after treatment as usual, *F* = 5.78, *p* .019, *r* .29. Two participants withdrew due to strenuous testing and one due to adverse effects.

**Conclusions:**

GMT had clinically reliable and lasting effects on subjective executive function. The intervention is a valuable addition to available treatment with considerable gains at low cost.

**Trial registration:**

Registered at clinicaltrials.gov NCT03048695 09/02/2017.

## Introduction

Executive functioning (EF) is important for education, work and social functioning [1]. EF is a set of interrelated higher-order mental processes involving top-down control of cognition, emotion and behavior necessary for successful navigation of complex everyday situations [2]. Definitions of EF include the core components of inhibition, shifting (also known as set-switching or mental flexibility) and updating of working memory, as well as more complex processes such as planning and problem solving [3, 4].

Executive functioning is among the most consistently impaired cognitive domains in schizophrenia spectrum disorders on tests of inhibition, shifting and planning, as well as manipulation and maintenance of working memory [5]. Compared to healthy controls, persons with schizophrenia also report significantly more complaints of EF difficulties in everyday life on the Behavior Rating Inventory of Executive Function – Adult version (BRIEF-A) [6, 7].

EF impairments are also found among persons with psychosis risk [8]. Psychosis risk syndromes include attenuated positive symptoms, brief intermittent psychotic symptoms and genetic risk combined with deteriorated functioning [9, 10]. Emerging evidence suggests that cognitive remediation in early intervention for psychosis could potentially have a preventative effect on the burden of illness through preserving cognition and everyday functioning [11–14]. However, there is a lack of evidence for the efficacy of cognitive remediation in psychosis risk syndromes at present [15].

Lower scores on objective measures of EF (neuropsychological tests) predict poorer everyday functioning, greater need for vocational support and poorer life satisfaction in schizophrenia spectrum disorders and psychosis risk syndromes [16–20]. Fewer subjective EF complaints on the BRIEF-A is associated with greater personal recovery in schizophrenia spectrum disorders [21].

Goal Management Training (GMT) is a metacognitive strategy training program that aims to improve EF [22, 23]. Metacognitive strategy training is a mode of cognitive remediation that involves top-down learning of a mental strategy, rather than bottom-up learning through repetition of tasks. The strategy training promotes awareness of cognitive deficits, and facilitates increased self-monitoring and control over mental processes [24]. Metacognitive strategy training should not be confused with metacognitive training, which targets bias in thought content, or metacognitive therapy which targets rumination and worry [25]. Due to the complexity of interacting executive functions, metacognitive strategy interventions are recommended for EF impairments [26]. GMT has proved effective in people with different neurological and mental disorders [24]. The theory behind GMT posits that failures in goal-directed behavior often are due to lapses in sustained attention [27]. For example, one of our participants complained that if she were interrupted by the sight of a bill while vacuuming, she would forget to finish vacuuming. Instead, she would pay the bill, get caught up watching videos on the computer, and return later to find the vacuum cleaner in the middle of the room. Such distracted behavior with sudden bursts of activity is a hallmark of executive dysfunction and is often a sign that goal-directed behavior has been replaced by habits *(“When I am on the computer, I watch videos”*) or reliance on cues in the surroundings (seeing the bill or the vacuum cleaner) [27]. GMT teaches participants to replace automatic, distracted behavior and instead set, prioritize, maintain and perform goals through verbal self-instructions (Table 1).

**Table 1:** Functions of the steps in the GMT strategy

1. Stop	Interrupting automatic behavior
2. Focus on your breath	Adjusting arousal, present-mindedness
3. Define your goal	Forming and prioritizing task goals
4. Check the mental blackboard	Updating of working memory
5. Divide the goal into subgoals	Chunking of information
6. Check what you are doing	Task- and self-monitoring

Several cognitive remediation studies for individuals with schizophrenia spectrum disorders include training in metacognitive strategies in combination with drill and practice or vocational training [28–32]. However, few studies appear to have assessed the effect of a stand-alone metacognitive strategy training on EF in schizophrenia spectrum disorders and none in psychosis risk syndromes [15, 33]. Studies of stand-alone interventions are important to understanding mechanisms behind change in cognitive remediation. Furthermore, most studies have focused on mental strategies tailored to specific individuals or situations. GMT, in contrast, offers guiding principles that can be applied across any number of everyday activities [22, 34]. In addition, GMT is a manualized group intervention that can be administered in only nine sessions. Therefore, GMT could potentially prove to be an easy to implement, cost-effective intervention with a broad impact on everyday functioning [34]. GMT has been introduced for people with schizophrenia with promising results in one case-study and a recent randomized controlled trial (RCT) that combined GMT with occupational therapy [35, 36]. The individual from the case study showed better performance of familiar and novel real-life tasks after intervention. The effects remained after 2 years and he also reported increased self-confidence in performing activities of daily living [36]. The RCT that combined GMT with occupational therapy was aimed at adults with treatment resistant schizophrenia. The participants in the treatment group showed greater improvements in activities of daily living scored by observers [35].

The aim of the present RCT is to determine the effectiveness of GMT on executive functioning in a sample of young participants with early schizophrenia or psychosis risk. The potential of GMT for improving daily life functioning, symptoms of psychosis and well-being is also explored. A recent master thesis investigated the effects of GMT on measures of wellbeing among participants with a diagnosis in the schizophrenia spectrum in the sample and found that GMT significantly improved self-efficacy, but not self-esteem or quality of life [37]. The present study reports the effect of GMT on subjective EF (self-reported) and objective EF (neuropsychological tasks), symptoms of psychosis, functional capacity and daily life function.

Based on previous GMT research, we hypothesized improved subjective and objective EF following GMT [24]. As GMT is a metacognitive strategy training program, it might be expected to have the largest impact on EF in real-world situations [22]. Thus the trial was powered to detect meaningful differences on the primary subjective outcome measure, the BRIEF-A questionnaire. A computerized test of inattentiveness, Connors Continuous Performance Test (CPT3) [38] was chosen as a primary outcome measure for objective EF because it has been sensitive to change in previous GMT studies [39]. Given the close link between EF and everyday functioning in schizophrenia and psychosis risk syndromes, we further hypothesized improved functional capacity and independent living [40–42]. Even though cognitive remediation for schizophrenia does not target psychotic symptoms, small reductions in symptoms have been seen across previous studies [43]. Cognitive remediation appears to be especially beneficial for the reduction of negative symptoms [44]. Moreover, associations have been found between poor objective EF performance and negative and disorganized symptoms, but not positive symptoms [45, 46]. Thus, we hypothesized a reduction in negative and disorganized symptoms following GMT.

## Methods

### Participants

Eighty-one participants, 49 males (60%) and 32 females (40%), were recruited among patients referred for treatment of psychosis at a regional, public hospital, Innlandet Hospital, in Norway 2017–2020. The majority of participants were recruited through the hospital’s specialized early detection and intervention for psychosis clinics, resulting in a young sample between the ages of 16 and 44. Mean age was 25 years (SD 6.35), and 94% of participants were between 16 and 35 years old. Sixteen individuals, aged between 18 and 40 with a mean age of 23 years, were diagnosed with psychosis risk syndromes. The remainder of the sample were diagnosed with a disorder in the schizophrenia spectrum. See Table 5 for further details of participant characteristics.

The inclusion criteria were age (16 to 69 years), diagnosis (schizophrenia spectrum disorder according to the criteria in the Diagnostic and Statistical Manual of Mental Disorders, DSM-IV [47] or psychosis risk syndrome [9, 10]) and self-reported executive dysfunction (Total *T*-score above 55 on the BRIEF-A, considered clinically relevant in the Norwegian context [6, 48]). Exclusion criteria included comorbid neurological conditions, ongoing alcohol or substance abuse, intellectual impairment (IQ < 70) and treatment for psychosis for more than 5 years.

The study was preregistered at clinicaltrials.gov (NCT03048695 09/02/2017). Due to time consuming and strenuous assessment days, the assessment protocol was reduced after pre-registration so that some measures were only collected at baseline including the Iowa Gambling Task [49] and Letter Number Sequencing Test from WAIS-IV [50]. Goal Attainment Scale [51] was used only in the intervention group as it was integrated into the GMT-manual. The everyday functioning questions were simplified. The Cognitive Failures Questionnaire [52] was left out of the protocol due to an administration error.

The study was approved by the Regional Committee for Medical and Health Research Ethics Norway (2015/2118), and conducted in accordance with the Helsinki declaration. Informed consent was obtained for all participants. Advisers with service-user experience employed by the hospital were consulted during the planning and execution of the study. For instance, they advised on recruitment procedures and adaption of the intervention for a new patient population. An adviser also observed one of the first GMT-sessions gathering feedback from participants.

### Procedure

Participants were assessed for diagnostic eligibility by a trained psychologist using the Structured Clinical Interview for the Diagnostic and Statistical Manual of Mental Disorders-IV (DSM-IV) Axis I disorders, SCID-I and Structured Interview for Prodromal Symptoms [9, 53]. Symptoms of psychosis were assessed with the Structured Clinical Interview for the Positive and Negative Syndrome Scale for Schizophrenia, the SCI-PANSS [54]. Symptoms were grouped according to a five-factor consensus model with positive, negative, disorganized, depressed and excited symptoms [55].

**Fig. 1:**
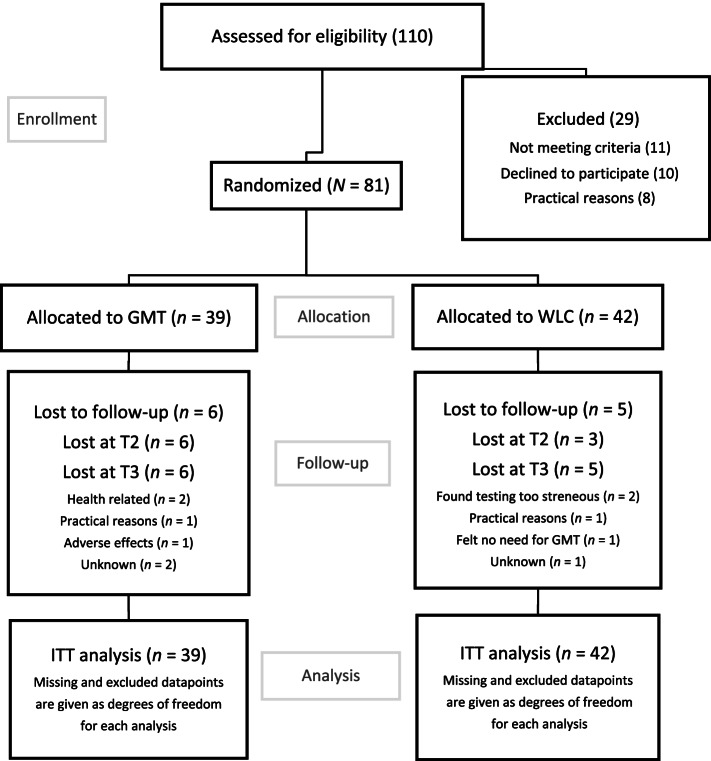
Flowchart of allocation and attrition

Figure 1 is a flow chart of participation [56].

Participants were randomly assigned in a parallel group trial design to either GMT (*n* = 39) or a Wait-List Control condition (WLC; *n* = 42) by a person independent from the study team using computer-generated random assignment from https://www.randomizer.org. Trained clinicians undertook baseline assessments (T1), post-treatment assessments immediately following GMT completion (T2 at 5 weeks) and follow-up assessments 6 months after GMT completion (T3 at 30 weeks). Conditions were masked from the raters gathering post-intervention and follow-up assessments. The raters were ordinarily employed in a ward separate from the intervention sites both in terms of organization and geography. To our knowledge, no instances of unmasking occurred. Participants received GMT in addition to treatment at usual for psychosis according to Norwegian national guidelines [57]. Treatment frequently involved a combination of medication and psychotherapy. Participants with psychosis risk syndromes received treatment for sub-threshold psychotic or general symptoms where indicated, but did not receive antipsychotics [57]. The control group members were offered GMT after follow-up assessment. The trial ended when a sufficient number of participants had been recruited.

### Intervention: Goal Management Training

Goal Management Training was administered to small groups of participants in nine, 2-h sessions (twice weekly). All sessions were held by the same clinical psychologist trained in GMT by a specialist in neuropsychology and GMT methodology, together with a local co-therapist. Co-therapists were given basic training in GMT and were doctors, psychologists, psychiatric nurses or occupational therapists. The training followed a script with accompanying PowerPoint slides and participant workbooks. Participants received a daily text message prompting strategy use between sessions four and nine [58, 59]. The current GMT-manual includes mindfulness breathing exercises to encourage adequate arousal and further improve the focus on goals in the present situation [27, 60]. The Norwegian translation of the GMT-manual [39] used in previous studies was revised by removing a mindfulness exercise involving sensory scanning of the body to reduce discomfort in case of tactile hallucinations or anmalous self-experiences. An exercise was added where participants developed one individual long-term goal according to the procedures in goal attainment scaling, because a review of GMT studies showed that personal goals increased effect of the intervention [51, 61]. Between-session assignments were reduced from three to two exercises due to the frequency of sessions (twice a week). Examples of assignments between sessions were collecting personal examples of inattentive slips, practicing mindful breathing or rehearsing the strategy for 30 min per day. See Table 2 for content of GMT.

**Table 2:** Content of Goal Management Training

Module	Content	Demonstrations	Assignments at home
1. Present- and absentmindedness	Absentmindedness is normal. Present mindedness can be practiced.	Clapping task demonstrating inattention Mindful eating of a raisin	Record absentmindedness, practice present mindedness
2. Slip-ups	Absentmindedness can lead to slip-ups	Clapping task Set personal goal	Record slip-ups, practice present mindedness
3. The autopilot	Acting on autopilot can lead to slip-ups	Sorting cards Breathing exercise	Record slip-ups, practice breathing exercise
4. STOP the autopilot	Saying STOP interrupts the autopilot and allows refocus	Sorting cards with STOP Short breathing exercise	Practice STOP 30 min daily, practice breathing exercise
5. The mental blackboard	Update working memory using the STOP-FOCUS-CHECK sequence	Sorting cards with distraction Short breathing exercise	Practice strategy 30 min daily, practice breathing exercise
6. State the goal	Stating goals aloud before and during tasks helps goal-attainment	Complex exercise that requires switching between 5 tasks	Practice strategy 30 min daily
7. Decision making	Recognize stress as a signal to use strategy to overcome indecision	Complex decision making task	Practice strategy when needed, internet shopping task
8. Dividing goal into subgoals	Large tasks are often made up of smaller tasks. Use subgoals when overwhelmed.	Define the subgoals in moving house and plan a wedding	Practice strategy when needed, internet shopping task
9. Check if goal is met	Checking if current actions are helpful in reaching the goal	Revisit clapping task Summary of the training	

Because the metacognitive strategy is gradually taught by adding steps from session four to nine, all sessions should be attended in order. Therefore, individual sessions were offered in cases of absence. The 33 participants in the intervention group who completed all three assessments points attended all nine sessions. Five participants completed the last three sessions via videoconference due to the outbreak of the Covid-19 pandemic.

### Measures

An overview of all measures reported in this study is provided in Table 3. The pre-registered primary outcome measures were BRIEF-A (subjective EF), CPT3 (objective EF) and SCI-PANSS (symptoms of psychosis).

**Table 3:** Measures

Instrument	Outcome variables	Time points
**SCID-I**: Diagnostic and Statistical Manual of Mental Disorders-IV (DSM-IV) Axis I [53]		Inclusion
**SIPS**: Structured Interview for Prodromal Symptoms [9]		Inclusion
**SCI-PANSS:** Structured Clinical Interview for the Positive and Negative Syndrome Scale for Schizophrenia [54]	Positive, negative, disorganized, depressive and excited symptoms	T1, T2, T3
**SCL-10**: Symptoms Check List [62]	Total score	T1, T2, T3
**WASI:** Wechsler Abbreviated Scale of Intelligence [63]	Estimated IQ from Matrix Reasoning and Vocabulary	T1
**WAIS-IV:** Wechsler’s Adult Intelligence Scale, 4th edition [50]	or Estimated IQ from General Ability Index (GAI)	T1
**BRIEF-A:** Behavior Rating Inventory of Executive Function - Adult version [6]	Total raw score 9 subscale scores	T1, T2, T3
**CPT3:** Conners Continuous Performance Test 3rd edition [38]	Detectability (d’) raw score	T1, T2, T3
**Digit Span** from the Wechsler Adult Intelligence Scale – 4th edition, WAIS-IV [50]	Total number of correct trials from the forward, backwards and sequential conditions	T1, T2, T3
**CWIT:** Color-Word Interference Test from the Delis-Kaplan Executive Function System (D-KEFS) [64]	Time – raw scores in seconds Raw contrasts between conditions 4 versus 3 (switching) and between condition 3 versus 1 and 2 (inhibition)	T1, T2, T3
**Tower** task from D-KEFS [64]	Total achievement score	T1, T2, T3
**UPSA-B:** University of California San Diego Performance-based Skills Assessment, brief version [65, 66]	Total score on the finance and communication modules	T1, T2, T3
**Hotel Task** [59]	Time expressed in number of seconds deviating from optimal time distribution between five tasks	T1, T2, T3
**SFS:** Social Functioning Scale [67]	The scores from the subscales Independence Competence and Independence Performance	T1, T2, T3
**GAF:** Global Assessment of Functioning-Split Version [47]	GAF-F global function score	T1, T2, T3

*Abbreviations*: *T1* Time one, baseline assessment (0 weeks), *T2* Time two, post-intervention assessment (5 weeks), *T3* Time three, follow-up assessment 6 months after intervention (30 weeks)

*Subjective EF* in everyday situations was measured using the 75-item questionnaire BRIEF-A [6]. The instrument has shown good test-retest reliability ranging from *r* .82 to .93 across nine subscales [6]. The scale showed good internal consistency in the present study at baseline with an adequate Cronbach’s Alpha score of α .95 for the total score.

*Objective EF* was assessed with the following tests: Inattentiveness was measured with the Conners Continuous Performance Test - 3rd edition (CPT3) [38]. The raw score for detectability (d’) analyzed is a signal-to-noise ratio that captures ability to correctly respond to targets while inhibiting responses to non-targets. Higher scores indicate poorer performance. The measures are reported to have adequate split-half reliability in a normative sample *r* .92 (*r* .95 for those under 18 years) and test-retest reliability, *r* .74 [38].

Total score on the Digit Span task (forwards, backwards and sequential conditions) from the Wechsler Adult Intelligence Scale – 4th edition, WAIS-IV [50] was used to assess working memory. The test has adequate internal consistency in normative samples with Cronbach Alpha scores of α .84 in the forwards condition, α .78 in the backwards condition and α .89 in the sequential condition [50]. Test-retest reliability ranges from *r* .71–.77 across the three conditions [50].

Inhibition and shifting were assessed with the Color-Word Interference Test (CWIT) from the Delis-Kaplan Executive Function System (D-KEFS) [64]. The test-retest reliability correlations for the four conditions in a normative sample were CWIT1 Color naming, *r* .86, CWIT2 Word reading *r* .49, CWIT3 Inhibition *r* .71 and CWIT4 Inhibition/Switching *r* .52 in the age group 20–49. Among those under 19 years the correlations ranged from *r*. 77 to *r* .90 [64]. In the present study, two raw contrast scores for inhibition and shifting were used as outcome measures to separate out the confounding effects of processing speed [68, 69]. A contrast measure of inhibition was created by subtracting the average amount of seconds spent on CWIT1 and CWIT2 from CWIT3. A contrast measure of shifting was created by subtracting time spent on CWIT3 from time spent on CWIT4. Higher contrast scores indicate greater difficulties with inhibition and shifting.

Strategic planning was measured with the total achievement score from the Tower task from D-KEFS [64]. The total achievement score reflects the building of correct towers with as few moves as possible, requiring the ability to plan more than one step ahead. Higher scores indicate better performance. Test-retest reliability in a normative sample was *r* .41 (*r*. 51 for those under 18) [64].

Raw scores on the above neuropsychological tests were converted to *z*-scores, reversed where appropriate and averaged for a total mean score of objective EF. Positive mean scores indicated better performance.

*Symptoms of psychosis* were measured using SCI-PANSS [54]. The instrument was scored by a trained clinician and included a structured interview with participants, input from someone who knew the participant well and saw them regularly (e.g., a family member or treating clinician) and observations made during the interview. Thirty items were scored on a scale ranging from 1 (absent) to 7 (extreme). Items referring to hallucinations and delusions with a score higher than 4 (moderate) indicate psychosis. The instrument has shown adequate reliability in both in- and outpatient settings [70]. In the present study, items were grouped according to a five-factor consensus model [55]. The total scores for positive, negative, disorganized, depressed and excited symptoms were used as outcome measures.

A brief, ten-item version of the Symptom Check List, SCL-10, was used to assess self-reported psychological distress [62]. The SCL-10 has shown adequate psychometric qualities equivalent to longer versions of the instrument and it has been validated in the Norwegian population [71]. The questionnaire reflects subjectively experienced anxiety and depressed mood. Items are scored on a scale ranging from 1 (a little bothered) and 4 (very bothered). The total score from the questionnaire was used as an outcome measure.

*Functional capacity* measures included the brief version of the University of California San Diego Performance-based Skills Assessment, UPSA-B [65, 66] and the Hotel Task [59]. From the UPSA, the total score out of 100 for the Finance and Communication modules was used. The UPSA is a role-playing task imitating activities of daily life including paying a bill and making a telephone call. Higher scores indicate better performance. During the Hotel Task participants are instructed to divide their time equally between five different tasks: Sorting coins, proof reading, creating invoices, using a telephone directory and sorting names alphabetically. The number of seconds deviating from optimal time distribution between the five tasks was used as the outcome measure.

*Activities of daily living* was assessed with two subscales from the self-reported Social Functioning Scale, SFS [67]. The Norwegian translation of the scale has been shown to be reliable and valid among people with schizophrenia [72]. The two subscales Independence Competence and Independence Performance were considered the most relevant outcome measures [73]. The internal consistency of the Independence Performance subscale, α .81, and Independence Competence subscale, α .65, was adequate. Furthermore, global function was assessed with clinician ratings of the Global Assessment of Functioning - Split version, GAF-F [47]. The scale ranges from 0 to 100 and higher scores indicate better functioning across important areas of life such as school or work, socially and at home. Ratings have been shown to be consistent among experienced raters [74].

### Data analysis

Analyses were performed using SPSS, version 26. In order to describe EF at baseline, one-sample *t*-tests were run comparing normed scores from the sample to standardized normative means gathered from the test manuals of EF instruments. Main outcome analyses were run using raw scores to retain variance. Outliers more than three standard deviations from the mean or with extreme residuals were excluded. The scores for CWIT Inhibition and SFS Independence Competence were log transformed to account for skewed distributions of scores. Group comparisons at baseline between GMT and WLC, and between completers and non-completers, were done using the Mann-Whitney Test for continuous variables and Pearson Chi Square for categorical variables.

A-priori power calculations based on existing GMT-studies indicated that to detect an effect size on the primary outcome measure of *r*. 30 (*d* 0.65), a sample size of *n* = 60 would be sufficient to render power of 80% with the alpha level set to *p* .05. Based on the principle of intention-to-treat (ITT), available data for all 81 participants were entered into a linear mixed model analysis for repeated measures [75]. Missing data were assumed to be missing at random. Group, time and group by time interactions were assessed as fixed effects and *p*-values < .05 were considered statistically significant. A first-order autoregressive covariance matrix was chosen for the repeated measures. Random subject intercepts were allowed for. Post-hoc explorations of change within treatment groups were done by running the models separately for each group.

As a precaution, age, sex, years of education, diagnosis, symptoms and treatment content in TAU (drug therapy and psychotherapy) were added one-by-one as co-variates in the mixed model analysis to control for potential influence on significant group x time interactions.

Effect sizes were expressed as Pearson’s *r* for the group x time interaction effects:

$$r=\sqrt{\frac{F_{\left( time\ x\ group\right)}\ }{F_{\left( time\ x\ group\right)}+ Df}}$$

Reliable Change Index (RCI) was calculated for the primary outcome measure that showed a significant interaction effect, BRIEF-A, to identify individuals with clinically reliable improvement from baseline (T1) to follow-up (T3) [76].

## Results

### Baseline characteristics of the sample

At baseline, the sample showed significantly more subjective complaints of EF with a mean total *T*-score of 68 on BRIEF-A when compared to normative samples [6]. The sample showed comparable performance to normative samples on the Digit Span test. All conditions of the CWIT were performed slower than the normative average, but there was no additional speed reduction on the conditions requiring shifting and inhibition, similar to previous studies [69]. The sample did, however, have more difficulty differentiating between targets and non-targets on the CPT3. Table 4 shows the executive functioning in the sample compared to the standardized means derived from large norming samples with healthy participants listed in the test manuals of the instruments [6, 38, 50, 64].

**Table 4:** Executive functioning at baseline (*N* = 81)

	*Study Sample*		*Standardized norms*			
	*M*	*SD*	*M*	*SD*	*t*^*a*^	*p*
BRIEF-A: Total *T*-score	68.08	10.59	50	10	14.68	**< .001**
Inhibit *T*	58.43	11.72	50	10	6.19	**< .001**
Shift *T*	62.82	11.30	50	10	9.76	**< .001**
Emotional Control *T*	58.01	11.73	50	10	5.88	**.003**
Self-Monitor *T*	53.95	10.97	50	10	3.10	**< .001**
Initiate *T*	68.92	12.01	50	10	13.55	**< .001**
Working Memory *T*	67.73	10.38	50	10	14.70	**< .001**
Plan/ Organize *T*	62.53	9.83	50	10	10.96	**< .001**
Task Monitor *T*	61.01	11.01	50	10	8.61	**< .001**
Organization of Materials *T*	54.78	12.58	50	10	3.27	**.002**
Digit Span total Scaled Score	9.88	2.60	10	3	−0.39	.697
CWIT1: Color Naming SS	6.86	2.91	10	3	−9.57	**< .001**
CWIT2: Reading SS	8.05	3.20	10	3	−5.42	**< .001**
CWIT3: Inhibition SS	8.27	3.45	10	3	−4.46	**< .001**
CWIT4: Inhibition & switching SS	7.95	3.85	10	3	−4.46	**< .001**
CWIT Contrast Inhibition SS	11.41^b^	2.66	10	3	4.69	**< .001**
CWIT Contrast Shifting SS	9.73	2.97	10	3	−0.80	.429
Tower total achievement SS	10.50	2.36	10	3	1.90	.062
CPT3 d’ *T*-score	54.87	10.38	50	10	4.23	**< .001**

Bold values are statistically significant

^a^Results of one-sample t-tests compared to standardized means of normative samples from the manuals of the instruments [6, 38, 50, 64]

^b^Note that the scaled score for CWIT Inhibition is higher than the normative mean

### Group comparisons at baseline

Any baseline differences between the groups were considered incidental due to randomization [77]. The GMT-group reported more subjective EF complaints at baseline, *F*(1,72) = 6.66, *p* .012. The GMT-group also showed a significantly lower level of negative symptoms compared to the WLC-group, *F*(1, 79) = 17.34, *p* .008. The groups were otherwise comparable, see Table 5.

**Table 5:** Baseline characteristics of the randomized sample (*N* = 81)

	GMT (*n* = 39)				WLC (*n* = 42)				
	Frequency	*M*	*SD*	*SE*	Frequency	*M*	*SD*	*SE*	*P*
Sex									.102
Female	19 (49%)				13 (31%)				
Male	20 (51%)				29 (69%)				
Age		25.46	6.68	1.07		24.38	6.07	.94	.504
Years of Education		13.00	2.00	.32		12.81	1.67	.26	.814
Estimated IQ^a^		98.65	15.11	2.48		98.97	13.17	2.11	.670
Diagnosis									.869
Schizophrenia spectrum disorder^b^	31 (80%)				34 (81%)				
Psychosis risk syndrome^c^	8 (20%)				8 (19%)				
DUP^d^ (weeks)		205.44	266.77	42.72		185.93	210.11	32.42	.924
Hospitalizations		2.62	5.13	.82		2.88	4.27	.66	.463
Months in hospital		4.46	8.67	1.39		5.23	6.56	1.01	.287
Drug therapy	30 (77%)				30 (71%)				.573
Antipsychotics	23 (59%)				27 (64%)				.623

^a^Estimated IQ: General intellectual ability was estimated at baseline with Vocabulary and Matrix Reasoning subtests from the Wechsler Abbreviated Scale of Intelligence (WASI) [63]. A few participants had GAI (General Ability Index) scores from Wechsler’s Adult Intelligence Scale, 4th edition (WAIS-IV) in place of WASI scores [50]

^b^Classifications were schizophrenia (GMT *n* = 12, WLC *n* = 17), schizoaffective disorder (GMT *n* = 6, WLC *n* = 8), schizophreniform episode (GMT *n* = 4, WLC *n* = 2), delusional disorder (WLC *n =* 1) and psychosis not otherwise specified (GMT *n* = 9, WLC *n* = 6) [47]

^c^Classifications were positive symptoms syndrome (GMT *n* = 6, WLC *n* = 3), brief intermittent psychotic symptoms (GMT *n* = 2, WLC *n* = 3) and genetic risk combined with fall in function (GMT *n* = 0, WLC *n* = 2) [9]

^d^*DUP*: Duration of untreated psychosis defined as weeks from onset of psychotic symptoms until start of adequate treatment with antipsychotic medication or hospitalization in a specialized ward [78]

### Attrition

Nine subjects did not complete testing at T2, and this number increased to 11 at T3 making attrition 13.58% at the end of the study. There were no significant differences between completers and non-completers in demographical or clinical variables.

### GMT outcomes

A linear mixed model analysis showed a significant decrease in self-reported symptoms of executive dysfunction in everyday life in the GMT-group only, BRIEF-A Total score, *F*(1, 51.94) = 8.40, *p* .005, *r* .37. Results for subjective EF can be seen in Table 6. The result remained unchanged when controlling for age, sex, diagnosis, years of education, treatment and severity of psychotic symptoms. In particular, there was no main effect of negative symptoms on subjective EF, and adding the variable did not change the significant interaction effect between group and time on subjective EF. Of note, significantly more participants in the GMT-group (10 of 19, 52.60%) experienced reliable clinical change from baseline to follow-up on this measure compared to the WLC-group (2 of 18, 11.10%), χ2(1) = 7.27, *p* .007, φc .44 according to the RCI [76].

**Table 6:** Linear mixed model analysis (ITT *N* = 81): subjective executive functioning

	GMT Mean scores			WLC Mean Scores			Group x Time interaction					
	T1^a^	T2	T3	T1	T2	T3	*df*	*b* (GMT)	*b SE*	*95% CI*	*P*	*﻿r*
***BRIEF-A total***	149.34	140.30	132.55	136.60^b^	132.48	135.05	51.94	−7.62	2.63	−12.90, −2.35	**.005**	.37
*Inhibit*	15.29	15.35	14.73	13.54^b^	13.48	13.52	51.81	−0.72	0.37	−1.45, 0.02	**.056**	.26
*Shift*	11.97	12.45	11.41	11.33	11.79	12.57	44.16	−0.73	0.32	−1.38, − 0.07	**.030**	.32
*Emotional Control*	20.09	19.50	19.14	17.62^b^	17.38	18.00	47.36	−0.39	0.53	−1.46, 0.67	.463	.11
*Self-Monitor*	10.35	10.17	10.00	9.37	9.93	10.48	50.02	−0.66	0.25	−1.16, − 0.16	**.011**	.35
*Initiate*	18.49	17.90	16.45	17.35	18.22	18.11	51.82	−1.43	0.36	−2.15, −0.72	**< .001**	.49
*Working Memory*	17.12	17.21	16.38	15.67	16.00	16.38	53.14	−0.74	0.37	−1,49, 0.00	.051	.26
*Plan/ Organize*	19.89	20.25	18.09^c^	18.08^b^	19.76	19.90	63.41	−1.78	0.52	−2,83, −0.74	**.001**	.39
*Task-Monitor*	11.43	11.50	10.95	11.37	11.39	11.45	36.34	−0.35	0.33	−1.01, 0.31	.293	.17
*Organization of Materials*	15.21	15.90	15.05	13.95	14.34	14.48	48.12	−0.72	0.44	−1.59, 0.17	.109	.23

Bold values are statistically significant

^a^The time variable was coded 0 for baseline, 1 for post-intervention testing and 2 for follow-up

^b^The GMT-group had a higher mean score at baseline on the BRIEF-A total raw score and the subscales for Inhibit, Emotional Control and Plan/Organize

^c^Significant main effect of time

The results show no difference in effectiveness between the two groups measured with neuropsychological tasks. However, both groups improved significantly over time on the Tower task and in mean objective EF.

There were no significant differences between treatments in functional capacity, self-reported independent living or clinician ratings of global functioning. Both functional capacity and clinician rated function improved significantly over time in both groups.

Both treatment groups showed a reduction in positive, disorganized and excited symptoms over time, but no significant treatment effect of GMT were registered in psychotic symptoms assessed by a trained clinician with SCI-PANSS. The GMT-group experienced a significantly greater reduction in self-reported symptoms of anxiety and depressed mood measured by the SCL-10, *F*(1, 64.05) = 5.78, *p* .019, *r* .29. See Tables 7, 8 and 9 for results of the mixed model analyses.

**Table 7:** Linear mixed model analysis (ITT *N* = 81): objective executive functioning

	GMT *Mean scores*			WLC Mean Scores			Group x Time interaction					
	T1^a^	T2	T3	T1	T2	T3	*df*	*b* (GMT)	*b SE*	*95% CI*	*P*	*r*
***Objective EF mean***	−0.02^c^	0.18	0.22	0.02	0.21	0.38	70.43	− 0.05	0.05	− 0.15, 0.05	.331	.12
***CPT3 d’***	−2.79	−2.81	− 2.78	− 2.55	− 2.79	− 2.82	70.45	− 0.07	0.10	−0.27, 0.12	.463	.09
*Digit Span*	26.00	27.58	25.97	25.18	25.59	26.20	65.47	−0.54	0.49	−1.52, 0.44	.278	.13
*CWIT Inhibition*	29.36	30.81	29.15	26.49	27.07	25.53	48.53	0.03	0.05	−0.08, 0.13	.623	.07
*CWIT Switching*	6.68	5.68	6.48	6.95	7.89	6.14	58.65	0.03^d^	0.04	−0.04, 0.11	.391	.11
*Tower*	17.72^c^	18.21	19.58	18.21	20.10	20.68	84.07	−0.30	0.51	−1.33, 0.72	.558	.06
*UPSA*	78.03^c^	80.47	82.13	74.69	79.16	82.31	74.71	−1.66	1.16	−3.97, 0.65	.157	.16
*Hotel Task*	287.60^c^	321.79	263.48	389.32^b^	314.87	289.79	65.71	28.50	20.75	−12.93, 69.93	.174	.17

^a^The time variable was coded 0 for baseline, 1 for post-intervention testing and 2 for follow-up

^b^The GMT-group performed the Hotel Task significantly better than the WLC-group at baseline

^c^Significant main effect of time

^d^Variable was log transformed to correct skewed distribution of scores

**Table 8:** Linear mixed model analysis (ITT *N* = 81): self- and clinician rated functioning

	GMT Mean scores			WLC Mean Scores			Group x Time interaction					
	T1^a^	T2	T3	T1	T2	T3	*df*	*b* (GMT)	*b SE*	*95% CI*	*P*	*r*
**GAF-F**	43.87^c^	47.31	48.82	42.66	43.97	46.92	82.96	0.33	1.33	−2.31, 2.98	.802	.00
SFS:												
*Withdrawal*	8.07	8.85	9.75	7.97	7.39	7.80	49.97	0.44	0.35	−0.26, 1.14	.210	.18
*Interpersonal behavior*	6.85	6.05	6.70	5.67^b^	5.79	5.55	57.54	−0.15	0.30	−0.74, 0.44	.618	.07
*Pro-social activities*	12.71	11.58	14.32	10.36	9.89	8.68	34.94	0.84	0.81	−0.80, 2.48	.307	.17
*Recreation*	17.58	17.00	19.55	15.03	15.50	15.65	52.81	0.80	0.73	−0.67, 2.26	.280	.15
*Independence Competence*	33.21	34.26	34.84	31.55	31.56	31.21	54.35	0.17^d^	0.10	−0.03, 0.36	.097	.22
* Independence Performance*	24.59	25.16	26.75	23.18	23.46	24.15	48.73	0.91	0.74	−0.58, 2.40	.227	.17
*Employment*	6.16	5.55	6.75	5.54	4.43	4.80	51.60	0.47	0.45	−0.44, 1.38	.305	.14

^a^The time variable was coded 0 for baseline, 1 for post-intervention testing and 2 for follow-up

^b^The GMT-group scored significantly higher on the subscales Interpersonal behavior than the WLC-group at baseline

^c^Main effect of time

^d^Variable was log transformed to correct skewed distribution of scores

**Table 9:** Linear mixed model analysis (ITT *N* = 81): clinical symptoms

	GMT Mean scores			WLC Mean Scores			Group x Time interaction					
	T1^a^	T2	T3	T1	T2	T3	*df*	*b* (GMT)	*b SE*	*95% CI*	*P*	*r*
**SCI-PANSS**:												
*Positive*	12.21^c^	9.09	9.58	11.26	9.26	8.57	80.43	0.05	0.46	−0.85, 0.96	.910	.01
*Negative*	13.85	11.76	12.94	16.69^b^	15.87	15.00	78.36	0.35	0.60	−0.84, 1.54	.562	.07
*Disorganized*	6.87^c^	6.15	6.88	7.17	6.69	6.35	82.27	0.41	0.29	−0.17, 0.99	.165	.15
*Depressed*	10.38	8.52	8.61	10.36	9.97	9.54	75.45	−0.50	0.32	−1.13, 0.14	.123	.18
*Excited*	8.95^c^	7.67	8.18	8.38	7.08	6.92	85.37	0.34	0.35	−0.35, 1.03	.327	.11
**SCL-10**	24.87	20.45	22.00	23.44	23.59	24.25	64.05	−2.32	0.97	−4,25, −0.39	**.019**	.29

Bold values are statistically significant

^a^The time variable was coded 0 for baseline, 1 for post-intervention testing and 2 for follow-up

^b^The WLC-group had significantly higher mean levels of negative symptoms at baseline than the GMT-group

^c^Significant main effect of time

Post-hoc explorations of change within each group showed that the GMT-group demonstrated significant improvement on the primary outcome measure for objective EF, the CPT3, over time, *F*(34.18) = 4.33, *p* .045, *r* .33, while the WLC-group did not demonstrate statistically significant improvement, *F*(35.75) = 1.58, *p* .216, *r* .20. The GMT-group also showed significant improvement over time in self-reported performance (SFS Performance GMT *F*(21.07) = 5.17, *p* .034, *r* .44 versus WLC *F*(29.70) = 0.19, *p* .666, *r* .08) and competence in independent activities of daily living (SFS Performance GMT *F*(32.68) = 4.79, *p* .036, *r* .36 versus WLC *F*(22.73) = 1.39, *p* .251, *r* .24). However, improvement was significant in both groups for the Tower task, mean objective EF, GAF-F and the UPSA. None of the groups experienced improvement on the CWIT and, in fact, the WLC-group showed greater improvement on the Digit Span and the Hotel Task, *F*(40.40) = 9.82, *p* .003, *r* .44 compared to the GMT-group, *F*(25.02) = 1.92, *p* .176, *r* .26.

Post-hoc explorations of change within each group showed that the GMT-group demonstrated significant reduction in depressive symptoms, *F*(37.01) = 12.97, *p* < .001, *r* .51, while the WLC-group did not demonstrate statistically significant improvement, *F*(38.38) = 2.67, *p* .111, *r* .26. The opposite was found for excited symptoms. The WLC-group demonstrated a significant reduction in excited symptoms, *F*(41.80) = 12.01, *p* .001, *r* .47, while the reduction in the GMT-group did not reach statistical significance, *F*(44.51) = 1.78, *p* .189, *r* .20.

## Discussion

This study examined the efficacy of GMT in improving EF among people with schizophrenia spectrum disorders or psychosis risk syndromes. To our knowledge this is the first RCT of stand-alone GMT as an early intervention for this patient group. GMT led to a significant and clinically reliable reduction of dysexecutive problems in daily life 6 months after the intervention. The largest effects of GMT on self-reported EF were in initiating activities, planning/ organizing, self-monitoring and shifting focus between activities as assessed with the BRIEF-A subscales. The effect GMT had on increased initiation of activity is especially compelling as this has been reported to be the most impaired domain both in our sample and in a previous schizophrenia study [7]. Difficulty initiating activity is also a challenging symptom to treat in schizophrenia [79].

We did not find significantly greater improvement on objective EF measures in the GMT-group compared to the WLC-group. Since the post-hoc analysis showed improved scores on overall objective EF and the Tower task in both groups, this likely reflects practice effects due to repetition of measures similar to previous studies in schizophrenia [64, 80]. There could be several possible reasons why GMT changed subjective EF more consistently than objective EF. It may be that GMT primarily had a compensatory, rather than restorative, mechanism within the follow-up period of the present study [33, 81]. A restorative mechanism supposes an improvement in specific cognitive functions (for example through frequent task repetition) leading to improved performance on objective measures [82]. A compensatory mechanism supposes learning to use other, better functioning areas of cognition to work around specific challenges. Metacognitive strategy training programs such as GMT, could potentially have both a compensatory and a restorative effect [83]. The earliest effects of GMT might be expected at the behavioral level as a result of compensatory strategy use in real-world situations. However, there might also be a restorative effect of GMT on specific executive functions over time when the strategy becomes automatized through repetition. Current evidence implies that GMT leads to improved performance on neuropsychological tasks across study populations, especially in a working memory task [24]. It is not certain why the present study failed to show similar effects of GMT on objective EF. The study may have lacked sufficient power to detect small treatment effects, especially considering that our study sample performed as well as normative samples on some of the objective tasks at baseline [84]. It is also possible that people with psychosis require more support outside sessions in order to internalize GMT-strategies. Nonetheless, since GMT is a metacognitive strategy training rehearsed in real-world situations, neuropsychological tests may not have been the most suitable outcome measures in the present study. The end goal for GMT is improving goal-directed behavior in real life. The use of systematic observation of familiar and novel real-life tasks might hold the key to unlocking the real potential of GMT [22, 36].

Furthermore, subjective and objective measures of EF are rarely strongly correlated in neither healthy nor clinical samples [85, 86]. One of the main strengths of objective measures is limiting the influence of confounding factors through control over the test situation. As a consequence, the objective test setting provides too much structure to assess the complexity of interacting components of executive function required in real-life [87]. Subjective measures, on the other hand, are better at capturing complex everyday situations, but are more easily influenced by confounding emotional states [88]. Since the discrepancy between subjective and objective measures of cognition is often larger among persons with schizophrenia than in healthy samples, caution should be exercised in the interpretation of the mechanisms of change in subjective EF in the present study [89–92]. That is not to say that self-reported executive functioning is not of clinical importance as it has been shown to predict important life outcomes, for example academic performance in college [93], and impulse control in younger people [94]. In addition, fewer subjective cognitive complaints in schizophrenia are associated with better physical and psychological well-being [95]. Lower scores on the BRIEF-A in particular is associated with greater personal recovery among people with schizophrenia spectrum disorders [21]. Furthermore, it is possible that a reduction in executive difficulties in real-world situations leads to attempting more challenging tasks [21, 96, 97]. Over time this can build increased confidence in the mastery of activities of daily living, similar to what was observed in the first case study of GMT in schizophrenia [36].

There was no effect of GMT as a stand-alone intervention in functional capacity (UPSA and Hotel Task), self-reported activities of independent living (SFS) or clinician rated global functioning (GAF-F). Some of this may be due to methodological issues. For example, the UPSA may have lacked the sensitivity required to detect meaningful treatment effects, as it has shown ceiling effects in previous studies among younger individuals with a first episode of psychosis [98–100]. The Hotel task may have been subject to an inverse treatment effect due to similarities to a practical multi-tasking exercise during GMT. In a demonstration during session six, GMT-participants are instructed to shift quickly between tasks, but not divide their time equally as in the Hotel task. An inverse effect where GMT-participants perform more poorly on the Hotel task after GMT has been observed previously in a GMT study [27].

The post-hoc analysis of the SFS indicated that change did occur in self-reported performance and competence in activities of independent living in the GMT-group and not the WLC-group, but that the analysis lacked sufficient statistical power to reveal this in the main analysis. The clinician ratings of global function, however, showed that both groups improved their functioning over time showing that GMT did not outperform treatment as usual. Global function as defined in GAF-F is a very broad construct including areas of life not necessarily expected to change in the time span of the present study. Thus, using Goal Attainment Scale as an outcome measure of progress on individual goals of everyday functioning, as originally intended, would likely have been a more appropriate measure [101].

It is possible the interval of 6 months between intervention and follow-up measurements was not long enough to detect an effect of GMT on daily life function, since the GMT-strategy is internalized through repetition over time. Unfortunately, the present study did not assess the amount of strategy rehearsal each participant engaged in, and therefore it is not known to what degree the strategy was internalized. Nonetheless, our finding is in line with existing evidence indicating that cognitive remediation should be integrated into psychosocial rehabilitation programs in order to improve real-world functioning [11, 102, 103]. Combining GMT with restorative drill training and vocational rehabilitation may offer the most promise for achieving functional gains among people with psychosis and EF impairments [33, 102–104]. In a study by Vizzotto and colleagues GMT was combined with occupational therapy where participants with treatment-resistant schizophrenia practiced real-life tasks during sessions lasting a total of 45 h [35]. In that context, GMT improved performance on observed real-life tasks and informant reports of independent living. The aim of the present study was to assess stand-alone metacognitive strategy training as this has rarely been done in schizophrenia [15, 33]. However, the end goal of research on cognitive remediation in schizophrenia is to develop rehabilitation that maximizes the improvement in function, including participation in education and work [105, 106]. Future studies might consider comparing the effects of GMT to other forms cognitive training. Investigating GMT in combination with drill and practice training of executive functions might also further elucidate mechanisms and assist in the search for optimal treatments.

GMT led to improvement in self-reported, but not clinician rated, clinical symptoms. Since clinician rated symptoms were reduced over time in both groups, the reduction was most likely due to treatment as usual. It is possible that an improvement in EF would be associated with better self-regulation and a reduction in stressful experiences in daily life. A bidirectional interplay between EF and psychopathology has been suggested [107, 108]. Executive difficulties among adolescents and young adults with psychosis may exacerbate challenges in meeting the increased expectations of self-organization at home, in school, or in social situations. Failing to meet expectations from parents, peers, or teachers could cause stress and raise the risk of clinical symptoms [109, 110]. Accordingly, the reduction on self-rated symptoms of anxiety after GMT may be an expression of improved self-regulation and fewer stressful encounters. Perhaps it reflects those participants felt less overwhelmed in everyday situations when using the GMT strategies.

Converging evidence of reduced depressive symptoms after GMT from both self-reports and clinician ratings indicate that GMT had a positive effect on depression, as well. However, the change assessed by the clinicians was small and could only be detected in the post-hoc analysis. In addition, excited symptoms were only reduced in the control group, indicating that symptoms fluctuated in the sample over time and that the significant changes could be spurious findings.

The sample in the present study included both persons recently diagnosed with a schizophrenia spectrum disorder and persons with psychosis risk syndromes. Studies on cognitive remediation for psychosis risk are scarce and have not previously investigated metacognitive strategy training [15]. Some have argued that improved EF in everyday life could potentially protect against a worse prognosis by preserving role function during an important phase of life when work, social and family life begins to be established [11, 111]. In our sample, there were not enough participants with psychosis risk syndrome to analyze this subgroup alone. However, effects of GMT were similar in analyses with and without psychosis risk participants. Even though the current study cannot conclude that GMT has a preventative effect on prognosis, improvements in subjective EF among at-risk participants are important nonetheless because it may indicate a reduction of friction in everyday situations [112]. Everyday stressors tends to increase intensity of psychotic symptoms [113]. The relationship between cognition and stress in psychosis is in need of further elucidation [114]. However, improved self-reports of executive problems such as inattentiveness, impulsive behavior or challenges initiating activities may potentially have a protective effect early in psychotic illness [14, 21].

### Implications

GMT is a valuable addition to early intervention in the schizophrenia spectrum disorders and psychosis risk syndromes, because EF is important in everyday situations and frequently severely impaired in these patient groups [5, 7, 8]. Aside from the associations subjective EF has with personal recovery, experiencing that you are better able to plan, start and organize everyday tasks, monitor yourself and shift focus when required, could have a positive impact on the participants’ everyday life and adherence to treatment for psychosis. GMT proved to have clinically reliable and lasting effects after being administered in groups over a brief period of 5 weeks. Participants also reported less anxiety and depressed mood after intervention. Thus, this suggests that GMT can provide considerable gains at low cost in clinical settings. The standardized manual ensures fidelity and allows for efficient training of clinicians. Future studies should assess maintenance of strategies learnt during GMT [115].

### Strengths and limitations

The robust randomized design featuring masking of conditions and follow-up over 6 months with low attrition rates are important strengths of this study. The sample size ensured sufficient statistical power to detect moderate effects. The extensive assessment protocol with a multimodal approach to the measurement of EF is also a strength of the study. However, the protocol lacked observational measures of real-life situations and community functioning and was a missed opportunity of capturing potential beneficial effects of GMT on functioning [35, 36, 116]. The primary outcome measure that showed the largest treatment effect of GMT was self-reported EF, which may be vulnerable to cognitive deficits in self-evaluation, demand characteristics and social desirability bias [117]. The neuropsychological tests were the same at all assessment points. It would have been preferable to use tests with alternative versions to avoid practice effects.

An important question is whether the study has sufficient generalizability beyond this sample. The sample was young and had received treatment for psychosis for a maximum of 5 years or had psychosis risk syndromes. It is therefore somewhat uncertain if the results may be generalized to older adults who have been living with schizophrenia for a longer period of time. However, a recent study using the GMT protocol in combination with occupational therapy among adults with treatment-resistant schizophrenia and a higher mean age found similar results [35].

Treatment as usual at the time of participating in the present study varied somewhat since not all patients received both psychotherapy and drug treatment. This heterogeneity may have interfered with treatment effects of GMT. For example, attending psychotherapy may increase metacognitive capacity [96]. However, there were no significant difference between the GMT-group and WLC-group in concomitant treatment after randomization. Also, we did not find that other concomitant treatment moderated the effect of GMT when controlling for this statistically. Another caveat is that the GMT-group had fewer negative symptoms than the WLC-group. Since negative symptoms mediate the relationship between cognition and functional outcome, findings need to be replicated to ensure that the efficacy of GMT also applies to individuals with higher levels of negative symptoms [118]. Nonetheless, we did control for negative symptoms in the statistical analysis in the present study and negative symptoms did not influence the outcome of GMT on subjective EF.

Our sample was selected on the basis of EF complaints. In addition, the GMT-group reported greater difficulties with EF in everyday situations at baseline than the WLC-group, which may have inflated the effect size of the main finding, but the effect of GMT remained significant when controlling for this baseline difference by removing the main effect of treatment group [119].

## Conclusions

To our knowledge, this is the first high-quality RCT of stand-alone metacognitive strategy training in people with schizophrenia spectrum disorders and psychosis risk syndromes. Our main findings demonstrated that a five-week, group-based GMT program was effective in reducing self-assessed, daily-life executive dysfunction. Finally, the study had a low attrition rate, suggesting high participant acceptance of the intervention.

## Data Availability

The datasets generated and analyzed during the current study are not publicly available due to limitations of ethical approval involving the patient data and anonymity, but are available from the corresponding author on reasonable request.

## Abbreviations

GMT: Goal Management Training
EF: Executive functioning
RCT: Randomized controlled trial
WLC: Wait-List Control
T1: Time one, baseline assessment at 0 weeks
T2: Time two, post-intervention assessment after 5 weeks
T3: Time three, follow-up assessment 6 months after intervention (30 weeks)
BRIEF-A: Behavior Rating Inventory of Executive Function – Adult version
CWIT: Color Word Interference Test
CPT3: Conners Performance Test, 3rd edition
UPSA: University of California San Diego Performance-based Skills Assessment
SFS: Social Functioning Scale
GAF-F: Global Assessment of Function, Split version (Function)
